# Books Reviewed

**Published:** 1866-12

**Authors:** 


### Books Reviewed.

A Manual of the Principles of Surgery, based upon Pathology, for Students. By WM. CANNIFF, M. D., M. R. C. S., etc., etc. Philadelphia: Lindsay & Blakiston, 1866.

The author of this book excels in making familiar explanations of diseased processes, and has embodied the essentials of pathological surgery within the compass of his work. The student is hereby furnished with a condensed philosophy, both of disease and its modes, and means of cure; the physician is also supplied with the rationale of disease and treatment. Nutrition, development, growth, assimilation, decay and repair, and other similar topics first receive attention. Then inflammation and the diseases arising out of inflammation, congestion, stagnation, changes in the blood, serum, lymph, etc., etc. All the various topics connected with inflammation and its different modes of termination and the various means of cure, are fully and familiarly discussed, pathology, therapeutics, etc., receiving attention. In this volume of about four hundred pages is included a wide range of topics, and special diseases and accidents are treated with philosophical reasoning, such as cannot fail to attract and instruct. The illustrations are appropriate, and add to the value of the work; they are from Paget's Surgical Pathology. The author acknowledges the labors of others, and with becoming modesty speaks of those authors to whom he feels most indebted.

The work is given to the profession in the best style of the well known publishers and forms an attractive as well as instructive addition to the physician's library.

---

A Manual of Materia Medica and Therapeutics; being an abridgment of the late Dr. Pereira's elements of materia medica. By Frederic John Farre, M. D., Cantab, F. L. S., etc., assisted by Robert Bentley, M. R. C. S., F. L. S., Robert Warrington, F. R. S., F. C. S., and Horatio C. Wood, jr., M. D.

This work contains a succinct account of every article of the materia medica, and is very well arranged in every respect. The illustrations, which are two hundred and thirty-six in number, add very materially to the value of the work. We thus obtain not only a full account of drugs, but also a representation of the plant from which many articles of the materia medica are obtained. This work is designed for both practitioners and medical students, and will be found eminently suited to their wants. The sources from which medicines are obtained, physical characters, medicinal properties, modes of preparation, therapeutical indications, and dose, is all embodied in this book, and thus it will be seen how invaluable it is to both physicians and students. Our standard works upon Therapeutics still quote the opinions of those who have called attention to some article of medicine, and are often too enthusiastic in their opinions of its curative virtues. Physicians soon learn that nine-tenths of the articles used as medicines really add nothing to our means of curing disease, though this fact shows nothing further in this connection. They are to be known that we may understand how little many of them are worth, and are of course to be considered in works upon therapeutics. It is not, however, necessary to reiterate the opinions of authors forever and ever, many of whom were over-zealous in bringing to the attention of the profession new articles for the materia medica.

We should be glad to receive a work on Therapeutics, written upon the observation, and giving the opinions only of some man, who would give each article its just deserts. As at present constituted medical students start in practice, believing that medicine will do all that it is said to do, and find out slowly and after many disappointments that there is more promised than done.

Pereira's Materia Medica is, as every one knows, as much to be depended upon as any work upon the subject, and this condensation of it abridges, but does not lessen its value. It is bound in the most substantial manner, and is a very valuable addition to a physician's library.

---

A Guide to the Practical Study of Diseases of the Eye; with outline of their Medical and Operative Treatment. By JAMES DIXON, F. R. C. S., Surgeon to the Royal London Ophthalmic Hospital. From the third London edition. Philadelphia: Lindsay & Blakiston, 1866.

The recent rapid discoveries and improvements in ophthalmic science are so remarkable, that every physician at all interested in the progress of this department of his profession, eagerly peruses every new work offered the profession, expecting that progress and improvement has been the occasion of its appearance. Ophthalmology has been enriched by more discovery both in pathology and therapeutics within the last few years than any other branch of medicine or surgery, and so great changes have thus been made that the older authors are practically obsolete. The work before us is designed by the author to afford a guide to the study of diseases of the eye. He says: "In attempting this, I have chiefly described outward appearances, such as lie open to the view of the observer, and have said but little of those subjective symptoms which vary according to the peculiar susceptibility of individual patients."

We have taken great pleasure in careful perusal of this book, which, both in style and matter, is unsurpassed in any language. It does not claim to cover the whole field, but in plain and condensed form gives the essentials of ophthalmology as understood and practiced at the present time by the most distinguished and experienced observers. It embraces quite a wide range of topics, and furnishes a very valuable practical guide in the medical and surgical treatment of diseases of the eye.

---

Asiatic Cholera; a Treatise on its Origin, Pathology, Treatment and Cure. By E. WHITNEY, M. D., and N. B. WHITNEY, M. D. New York: M. W. Dodd. 1866.

This work on Cholera makes its appearance in an attractive and permanent style, and contains sections upon origin and development, progress and fatality, causes and propagation, pathology, phenomena or symptoms, modes of treatment, unsuccessful, physiological condition of the blood—non-æration or non-oxydation, maxims of rational practice, statistics, prophylaxis, remedies, etc., etc. The authors have collected from various reliable sources a great amount of statistical knowledge and incorporated into their work the opinions of many of the most reliable physicians, whose opportunities for observation are unsurpassed.

A great many monographs have been published upon cholera in view of its appearance in this country. Some have passed into the permanence of book form. The one before us possesses merits which entitle it to this distinction, and we are most happy to place it in our library as a valuable treatise upon Asiatic Cholera.

An Introduction to the Study of the Optical Defects of the Eye, and their treatment by the scientific use of Spectacles. By A. M. Rosebrugh, M. D., Toronto.

This pamphlet is a lecture introductory to a course on the diseases of the eye, which the author proposes to publish in pamphlet form, hoping they may be useful not only to his class, but to medical students generally. This lecture was read before the Canadian Institute, February 3d, 1866. It contains the philosophy of vision, its frequent deviations and the modes of treatment by the use of glasses, and constitutes an attractive and instructive lecture.

---

The Brain and Cranial Nerves, shewing their origin, arrangement, principal division and distribution. By Thos. S. Bulmer, under-graduate in medicine, Toronto University, late student of McGill University.

The above is the title of a large and comprehensive chart, which is designed to show more clearly to the student, and useful also to the practitioner, the origin and distribution of these several nerves. Although the matter contained in the chart is to be found in all text-books on the subject, yet by arranging them in this manner, it simplifies much this difficult part of anatomy. It is an idea deserving of recommendation, and we think all, especially students, cannot do better than by purchasing one of these charts.

